# Combined Treatment with Low Cytotoxic Ethyl Acetate *Nepenthes* Extract and Ultraviolet-C Improves Antiproliferation to Oral Cancer Cells via Oxidative Stress

**DOI:** 10.3390/antiox9090876

**Published:** 2020-09-16

**Authors:** Sheng-Yao Peng, Li-Ching Lin, Zhe-Wei Yang, Fang-Rong Chang, Yuan-Bin Cheng, Jen-Yang Tang, Hsueh-Wei Chang

**Affiliations:** 1 Department of Biomedical Science and Environmental Biology, PhD Program in Life Sciences, College of Life Science, Kaohsiung Medical University, Kaohsiung 80708, Taiwan; u107500026@kmu.edu.tw (S.-Y.P.); u106023020@kmu.edu.tw (Z.-W.Y.); 2 Department of Radiation Oncology, Chi-Mei Foundation Medical Center, Tainan 71004, Taiwan; 8508a6@mail.chimei.org.tw; 3 School of Medicine, Taipei Medical University, Taipei 11031, Taiwan; 4 Chung Hwa University of Medical Technology, Tainan 71703, Taiwan; 5 Graduate Institute of Natural Products, Kaohsiung Medical University, Kaohsiung 80708, Taiwan; aaronfrc@kmu.edu.tw (F.-R.C.); jmb@kmu.edu.tw (Y.-B.C.); 6 Department of Radiation Oncology, Faculty of Medicine, College of Medicine, Kaohsiung Medical University, Kaohsiung 80708, Taiwan; 7 Department of Radiation Oncology, Kaohsiung Medical University Hospital, Kaohsiung 80708, Taiwan; 8 Cancer Center, Kaohsiung Medical University Hospital, Kaohsiung 80708, Taiwan; 9 Center for Cancer Research, Kaohsiung Medical University, Kaohsiung 80708, Taiwan

**Keywords:** UVC sensitizer, Nepenthes, oral cancer, ROS, DNA damage

## Abstract

Ultraviolet-C (UVC) irradiation provides an alternative radiotherapy to X-ray. UVC sensitizer from natural products may improve radiotherapy at low cytotoxic side effects. The aim of this study is to assess the regulation for oral cancer cell proliferation by a combined treatment of UVC and our previously reported anti-oral cancer natural product (ethyl acetate extract of *Nepenthes adrianii* × *clipeata*; EANA). The detailed possible UVC sensitizing mechanisms of EANA such as effects on cell proliferation, cell cycle, apoptosis, and DNA damage are investigated individually and in combination using 3-(4,5-Dimethylthiazol-2-yl)-2,5-diphenyltetrazolium bromide (MTS) assay, flow cytometry, and western blotting at low dose conditions. In a 24 h MTS assay, the low dose EANA (5 μg/mL) and low dose UVC (12 J/m^2^) individually show 80% and combinedly 57% cell proliferation in oral cancer Ca9-22 cells; but no cytotoxicity to normal oral HGF-1 cells. Mechanistically, low dose EANA and low dose UVC individually induce apoptosis (subG1 accumulation, pancaspase activation, and caspases 3, 8, 9), oxidative stress (reactive oxygen species, mitochondrial superoxide, and mitochondrial membrane potential depletion), and DNA damage (γH2AX and 8-hydroxy-2′-deoxyguanosine). Moreover, the combined treatment (UVC/EANA) synergistically induces these changes. Combined low dose treatment-induced antiproliferation, apoptosis, oxidative stress, and DNA damage were suppressed by the ROS scavenger *N*-acetylcysteine. In conclusion, UVC/EANA shows synergistic antiproliferation, oxidative stress, apoptosis, and DNA damage to oral cancer cells in an oxidative stress-dependent manner. With the selective killing properties of low dose EANA and low dose UVC, EANA provides a novel UVC sensitizing agent to improve the anti-oral cancer therapy.

## 1. Introduction

Oral cancer exhibits high mortality and morbidity worldwide. The regular treatments for oral cancer include the surgery, chemotherapy, and radiotherapy [1]. Some oral tumors, especially in hypoxic conditions, are more resistant to radiotherapy since the therapeutic effect is reduced [2]. To reduce radioresistance in oral tumor therapy, a combined treatment of radiation and drug was proposed. In the past several drugs and natural products were combined with X-ray radiation to improve cell killing against cancer cells. For example, *Berberis amurensis*-derived berbamine [3] and cruciferous vegetables-derived sulforaphane [4] improve the radiosensitizing effect for head and neck cancer. A clinical drug gimeracil shows radiosensitizing effect for oral cancer cells [5].

In addition to ionizing X-ray, nonionizing radiation such as ultraviolet-C (UVC) was applied to anticancer treatment in addition to its bactericidal function. The benefit of UVC is that its generating device is cheaper and easy to set up. UVC was applied in experiments with pancreatic [6,7] and colon [8] cancer cells. Moreover, several drugs and natural products were combined with UVC to improve cell killing against cancer cells. For example, the combined treatment of low dose cisplatin (10 μM) and low dose UVC (10 J/m^2^) induces a synergistic inhibition of colorectal cancer proliferation [9]. The combined treatment of methanolic extracts of *Cryptocarya concinna* and UVC provides synergistic anti-oral cancer effects [10]. Accordingly, the development of more combined treatments of drugs or natural products with UVC warrants more detailed investigations.

*Nepenthes* plants are commonly used as herb medicine for cough, fever, and hypertension [11]. Certain *Nepenthes* extracts also inhibit microorganism growth [12,13,14] and inflammation [15]. Recently, we had reported the preferential killing effects of oral cancer cells by the treatment of extracts of *Nepenthes* plants, i.e., ethyl acetate extract of *Nepenthes adrianii* × *clipeata* (EANA) [16]. However, the radiosensitizing effect of EANA in combination with UVC has never been investigated.

The aim of this study is to assess the regulation of oral cancer proliferation by the combined treatment of the low dose UVC and EANA (UVC/EANA). Moreover, the detailed UVC sensitizing mechanism of EANA effects such as on cell proliferation, cell cycle, apoptosis, oxidative stress, and DNA damage are investigated using oral cancer cells for treatments.

## 2. Materials and Methods

### 2.1. Crude Extracts and Chemical

EANA was prepared and its ^1^H NMR spectrum was well characterized as described in our previous work [16]. Briefly, twigs and leaves of *N. adrianii* × *clipeata* were air-dried and soaked in methanol for extraction and further partitioned between ethyl acetate and water to produce EANA. The ^1^H NMR spectrum of EANA has been previously reported [16] and shows that plumbagin is the major bioactive component. *N*-acetylcysteine (NAC) (Sigma-Aldrich; St. Louis, MO, USA) was selected to be a oxidative stress scavenger for validating the influence of oxidative stress [17,18,19] by 1 h pretreatment of 10 mM [20].

### 2.2. Cell Cultures and Proliferation Determination

A human oral cancer cell line (Ca9-22) and normal oral cell line (HGF-1) were purchased from the Japanese Collection of Research Bioresources (JCRB) Cell Bank (National Institute of Biomedical Innovation, Osaka, Japan) and the American Type Culture Collection (Manassas, VA, USA), respectively. They were cultured in a humidified environment as indicated [21]. Cell proliferation (cellular metabolic activity) was determined by MTS assay containing tetrazolium compound (Promega Corporation, Madison, WI, USA) as described previously [22].

### 2.3. UVC Irradiation and EANA Treatment

After medium removal, cells were irradiated with a germicidal UVC lamp (254 nm) at the rate of 2 J/m^2^/s and exposed for 6 s [10] to achieve the necessary UVC dosages (12 J/m^2^). The concentration for EANA treatment in oral cancer cells was 5 µg/mL. For combined treatments, cells were firstly UVC exposed and post-incubated in EANA-containing medium for indication time as shown in figure legends. For the control treatment, cells were performed without UVC (the same procedures as UVC irradiation except for turning off the UVC lamp) and post-incubated in 0.025% DMSO-containing medium, which was the same DMSO concentration as EANA and EANA/UVC treatments.

### 2.4. Cell Cycle Analysis

7-Aminoactinmycin D (7AAD) (Biotium, Inc., Hayward, CA, USA) (1 μg/mL, 37 °C, 30 min) method was used to analyze cell cycle distribution [23] by a flow cytometer (BD Accuri C6; Becton-Dickinson, Mansfield, MA, USA) and FlowJo software (LLC, Becton-Dickinson). For Western blotting, primary antibodies recognized extracellular-signal-regulated kinase 1/2 (ERK1/2), the phosphorylated forms of ERK1/2, and p21 Waf1/Cip1 (12D1) (Cell Signaling Technology, Inc., Danvers, MA, USA), and p27 (C-19) (Santa Cruz Biotechnology; Dallas, TX, USA) (diluted 1:1000) were used to detect pathways for selected cell cycle inhibitors and ERK.

### 2.5. Apoptosis

Annexin V (Strong Biotect Corporation, Taipei, Taiwan)/7AAD [24] and pancaspase activity [25] methods were used to determine the apoptosis status by a flow cytometer (BD Accuri C6) analysis. For Western blotting, primary antibodies for the cleaved forms of PARP (Asp214), caspase (Cas) 8 (Asp391), Cas 9 (Asp330), and Cas 3 (Asp175) (Cell Signaling Technology Inc., Danvers, MA, USA) (diluted 1:1000) were used to detect protein expressions that indicate apoptosis. Primary antibody for mAb-β actin (Sigma-Aldrich, St. Louis, MO, USA) was used to detect loading control. A detailed procedure was described previously [26].

### 2.6. Intracellular ROS

2′,7′-dichlorodihydrofluorescein diacetate (DCFH-DA) at the condition of 10 μM, 37 °C, 30 min was used to detect the intracellular ROS content by a flow cytometer (BD Accuri C6) and applying its software [27].

### 2.7. Mitochondrial Superoxide (MitoSOX)

MitoSOX Red (Molecular Probes, Invitrogen, Eugene, OR, USA) at the condition of 50 nM, 37 °C, 30 min was used to measure the MitoSOX content by a flow cytometer (BD Accuri C6) and applying its software [28].

### 2.8. Mitochondrial Membrane Potential (MitoMP)

DiOC_2_(3) (Invitrogen, Eugene, OR, USA) at the condition of 5 nM, 37 °C, 30 min was used to measure the MitoMP by a flow cytometer (BD Accuri C6) and applying its software [29].

### 2.9. Antioxidant Gene Expressions

Trizol reagent (Invitrogen, Carlsbad, CA, USA) was used to extract total RNA, which was converted to cDNA by the OmniScript RT kit (Qiagen, Valencia, CA, USA) as described previously [30]. iQ SYBR Green Supermix (Bio-Rad Laboratories, Hercules, CA, USA) was used for quantitative RT-PCR (qRT-PCR). Antioxidant signaling genes [31,32,33], including NF-kB subunit (*RELA*), forkhead box O3 (*FOXO3*), superoxide dismutase 1 (*SOD1*), and heme oxygenase 1 (*HMOX1*) as well as the internal control gene *GAPDH* were detected by touch-down PCR program [34] using a MyiQ real-time machine (Bio-Rad). Their primer information is shown in Table 1. The relative mRNA level was analyzed by 2^−ΔΔ*C*t^ method [35] and expressed in log_2_ scale.

### 2.10. γH2AX

After fixation, the primary antibody for γH2AX (Santa Cruz Biotechnology; Santa Cruz, CA, USA) (1:500 dilution) was used at the condition of 1 h at 4 °C [40]. Secondary antibody conjugated with Alexa Fluor ^®^488 (Cell Signaling Technology) was mixed with 7AAD (5 μg/mL) for 30 min and analyzed by a flow cytometer (Guava^®^ easyCyte ^TM^; Luminex, TX, USA) and applying FlowJo software (LLC, Becton-Dickinson).

### 2.11. 8-Hydroxy-2′-Deoxyguanosine (8-OxodG)

After fixation, 8-oxodG was detected by antibody conjugated with FITC (1:10,000 dilution) (#8-OHdG Antibody (E-8): sc-393871 FITC; Santa Cruz Biotechnology, Inc.) at the condition of 1 h at 4 °C and analyzed by a flow cytometer (BD Accuri C6) and applying its software.

### 2.12. Statistics

One-way ANOVA with Tukey HSD post hoc test were applied to calculate the significance between multi-comparisons using JMP12 software (SAS Institute, Cary, NC, USA).

## 3. Results

### 3.1. UVC Sensitizing Responses of EANA in Oral Cancer Cells

**Figure 1:**
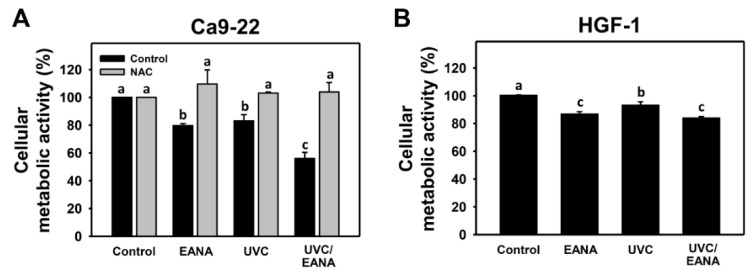
Cell proliferation of the ethyl acetate extract of *Nepenthes adrianii × clipeata* (EANA) and/or UV treatments for human oral cancer Ca9-22 and normal oral HGF-1 cells. (**A**,**B**) Cell metabolic activity was determined by MTS assay. With or without NAC (10 mM) pretreatment for 1 h, cells were post-treated with control (DMSO only), EANA (5 µg/mL), UVC (12 J/m^2^), and combined treatment (UVC/EANA) for 24 h. Treatments showing no overlapped labels (a–c) differ significantly between multiple comparisons (*p* < 0.05–0.0001). Data, mean ± SD (*n* = 3).

Low cytotoxic conditions at 80% oral cancer cell proliferation for UVC and EANA (12 J/m^2^ and 5 μg/mL) were selected (Figure 1A) to evaluate the UVC sensitizing response of EANA in combined treatment of oral cancer cells. Figure 1A also shows the combined treatment (UVC/EANA) displays lower proliferation at 24 h MTS assay for 57% than separate UVC, EANA, or control, suggesting that EANA has a UVC sensitizing ability to oral cancer Ca9-22 cells. Moreover, the antiproliferation effect of UVC and/or EANA on normal oral HGF-1 cells was evaluated. Figure 1B shows that UVC and/or EANA treatments in HGF-1 cells show less antiproliferation than that of oral cancer cells (Figure 1A).

We next examined the effect of NAC on the UVC sensitizing ability of EANA in antiproliferation of oral cancer cells. The antiproliferation effect of UVC and/or EANA against oral cancer cells were attenuated by NAC pretreatment (Figure 1A).

### 3.2. Cell Cycle Analysis of UVC and/or EANA Treatments in Oral Cancer Cells

**Figure 2:**
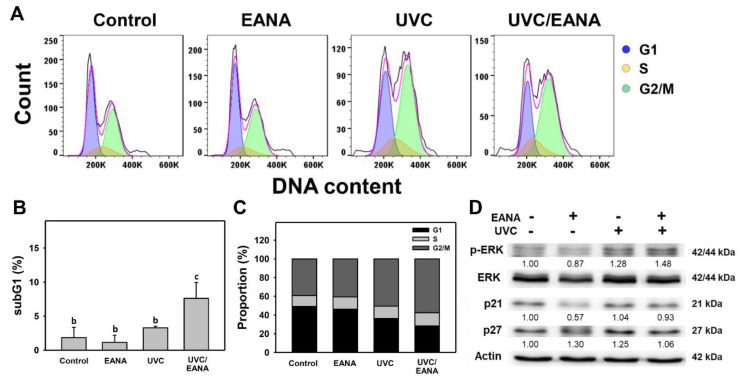
Cell cycle analysis of EANA and/or UV treatments for human oral cancer Ca9-22 cells. With or without NAC (10 mM) pretreatment for 1 h, cells were post-treated with control (DMSO only), EANA (5 µg/mL), UVC (12 J/m^2^), and combined treatment (UVC/EANA) for 24 h. (**A**) Typical flow cytometry patterns for cell cycle. (**B**,**C**) Statistical analysis for subG1 and cell cycle phases in Figure 2A. Treatments showing no overlapped labels (a–c) differ significantly between multiple comparisons (*p* < 0.05–0.0001). Data, mean ± SD (*n* = 3). (**D**) Western blotting analysis for p-ERK, ERK, p21, and p27. Intensity ratio for p-ERK was adjusted to ERK intensity. Intensity ratio for p21 and p27 was adjusted to actin intensity.

Figure 2A shows the DNA content profiles of oral cancer Ca9-22 cells after 24 h treatments with control (DMSO only), EANA (5 µg/mL), UVC (12 J/m^2^), or UVC/EANA. Figure 2B,C shows that both sub-G1 and G2/M populations (%) of the combined treatment (UVC/EANA) are significantly higher than other single treatments or control. In contrast, G1 populations (%) of UVC/EANA are significantly lower than other single treatments or the control.

ERK is a modulator for cell cycle regulation involving cell cycle inhibitors such as p21 and p27 [41]. The involvement for ERK, p21, and p27 in modulating cell cycle changes in UVC and/or EANA treated oral cancer cells were analyzed by Western blotting. Figure 2D shows that p-ERK overexpresses in UVC/EANA treatment compared to others. However, p21 and p27 show weak change between each treatment.

### 3.3. Annexin V/7AAD Changes of UVC and/or EANA Treatments in Oral Cancer Cells

**Figure 3:**
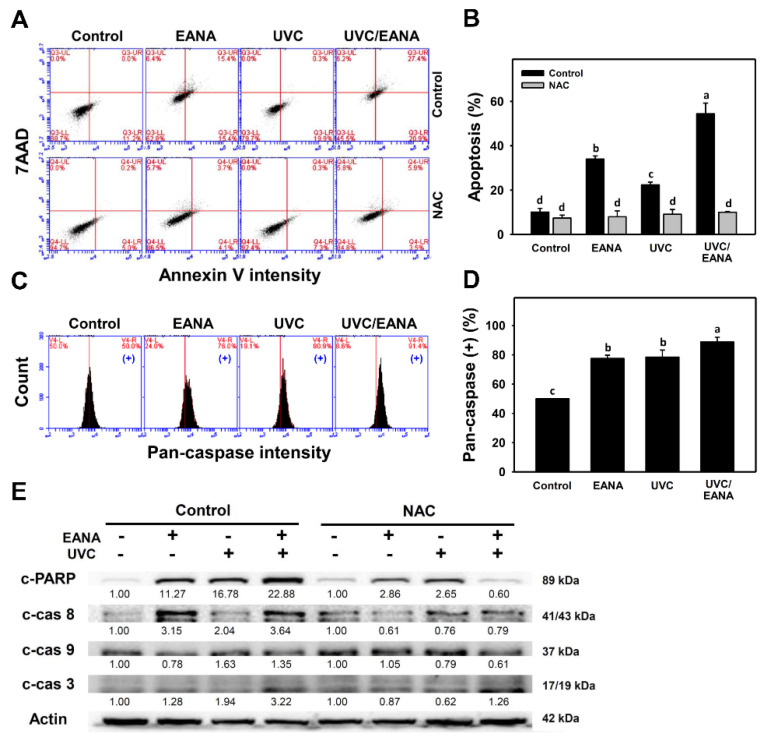
Apoptosis analyses of EANA and/or UV treatments for human oral cancer Ca9-22 cells. With or without NAC (10 mM) pretreatment for 1 h, cells were post-treated with control (DMSO only), EANA (5 µg/mL), UVC (12 J/m^2^), and combined treatment (UVC/EANA) for 24 h. (**A**) Typical flow cytometry patterns for the annexin V/7AAD method. Annexin V-positive regions were counted for apoptosis (%). (**B**) Statistical analysis for (**A**). (**C**) Typical flow cytometry patterns for pancaspase method. Pancaspase intensity-positive regions were marked as (+). (**D**) Statistical analysis for (**C**). Treatments showing no overlapping labels (a–d) differ significantly between multiple comparisons (*p* < 0.001–0.0001). Data, mean ± SD (*n* = 3). (**E**) Western blotting for apoptosis signaling proteins.

The subG1 accumulation (Figure 2) indicates an apoptosis-like event. The possibility of apoptosis was further validated by annexin V/7AAD assay at 24 h UVC and/or EANA treatments (Figure 3A). Figure 3B shows that the annexin V-positive (%) of the combined treatment (UVC/EANA) is significantly higher than other single treatments or the control.

We next examined the effect of NAC on UVC sensitizing ability of EANA by inducing apoptosis of oral cancer cells. The annexin V-detecting apoptosis of UVC and/or EANA against oral cancer cells were attenuated by NAC pretreatment (Figure 3B).

### 3.4. Caspase Activation of UVC and/or EANA Treatments in Oral Cancer Cells

To further confirm annexin V-detecting apoptosis in oral cancer cells after UVC and/or EANA treatments, the generic activation of pancaspases (caspases-1, 3, 4, 5, 6, 7, 8, 9) was detected (Figure 3C). Figure 3D shows that pancaspase-positive (%) of the combined treatment (UVC/EANA) is significantly higher than other single treatments or control.

The involvement of apoptosis signaling was further examined in Figure 3E. Cleaved forms of PARP (c-PARP), caspase 8 (c-Cas 8), and c-Cas3 expressions of the combined treatment (UVC/EANA) are higher than in other single treatments or the control. c-Cas 9 expressions are higher in UVC or UVC/EANA than others.

We next examined the effect of NAC on UVC sensitizing ability of EANA in inducing apoptosis signaling expressions of oral cancer cells. These inducible caspase signals of UVC and/or EANA against oral cancer cells were attenuated by NAC pretreatment (Figure 3E, right).

### 3.5. ROS Changes of UVC and/or EANA Treatments in Oral Cancer Cells

**Figure 4:**
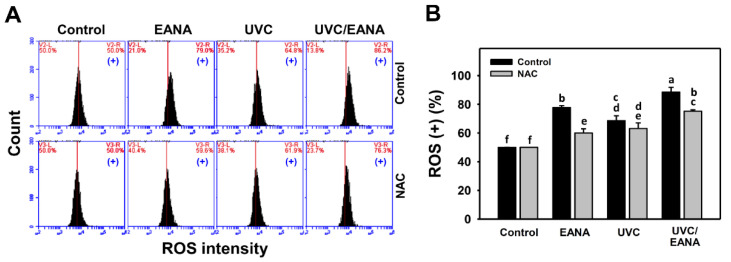
ROS analyses of EANA and/or UV treatments for human oral cancer Ca9-22 cells. Cells were treated with control (DMSO only), EANA (5 µg/mL), UVC (12 J/m^2^), and combined treatment (UVC/EANA) for 12 h. (**A**) Typical flow cytometry patterns for ROS content. ROS intensity-positive regions were marked as (+). (**B**) Statistical analysis for (**A**). Treatments showing no overlapping labels (a–f) differ significantly between multiple comparisons (*p* < 0.05–0.0001). Data, mean ± SD (*n* = 3).

ROS flow cytometry for oral cancer cells after UVC and/or EANA treatments was performed (Figure 4A). Figure 4B shows that ROS-positive (%) of the combined treatment (UVC/EANA) was significantly higher than other single treatments or control in a 12 h incubation.

We next examined the effect of NAC on UVC sensitizing ability of EANA in inducing ROS generation of oral cancer cells. The ROS generation of UVC and/or EANA against oral cancer cells was attenuated by a NAC pretreatment (Figure 4B).

### 3.6. MitoSOX Changes of UVC and/or EANA Treatments in Oral Cancer Cells

**Figure 5:**
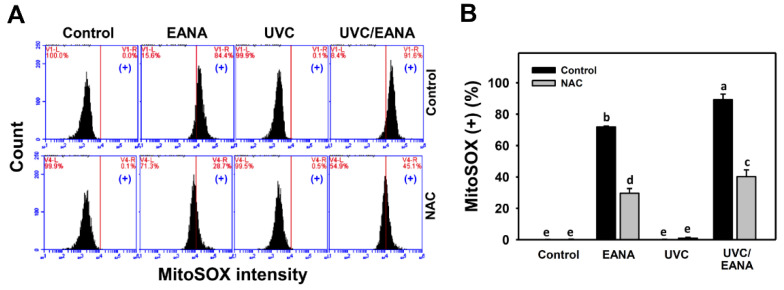
MitoSOX analyses of EANA and/or UV treatments for human oral cancer Ca9-22 cells. With or without NAC (10 mM) pretreatment for 1 h, cells were post-treated with control (DMSO only), EANA (5 µg/mL), UVC (12 J/m^2^), and combined treatment (UVC/EANA) for 24 h. (**A**) Typical flow cytometry patterns for MitoSOX content. MitoSOX intensity-positive regions were marked as (+). (**B**) Statistical analysis for Figure 5A. Treatments showing no overlapping labels (a–e) differ significantly between multiple comparisons (*p* < 0.001–0.0001). Data, mean ± SD (*n* = 3).

MitoSOX flow cytometry for oral cancer cells after UVC and/or EANA treatments was performed (Figure 5A). Figure 5B shows that MitoSOX-positive (%) of the combined treatment (UVC/EANA) is significantly higher than other single treatments or control for 24 h incubation.

We next examined the effect of NAC on UVC sensitizing ability of EANA in inducing MitoSOX generation of oral cancer cells. The MitoSOX generation of EANA only and UVC/EANA against oral cancer cells were attenuated by NAC pretreatment (Figure 5B).

### 3.7. MitoMP Changes of UVC and/or EANA Treatments in Oral Cancer Cells

**Figure 6:**
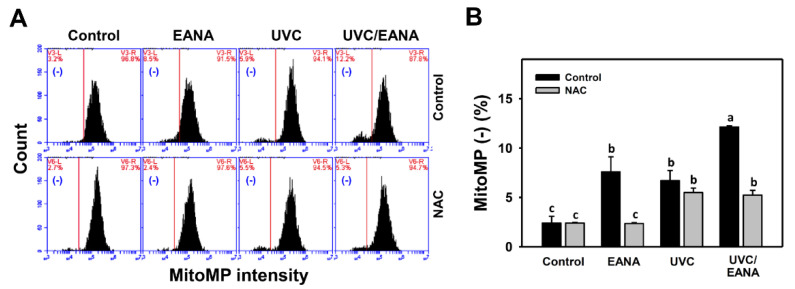
Mitochondrial membrane potential (MitoMP) analyses of EANA and/or UV treatments for human oral cancer Ca9-22 cells. With or without NAC (10 mM) pretreatment for 1 h, cells were post-treated with control (DMSO only), EANA (5 µg/mL), UVC (12 J/m^2^), and combined treatment (UVC/EANA) for 24 h. (**A**) Typical flow cytometry patterns for MitoMP content. MitoMP intensity-negative regions were marked as (−). (**B**) Statistical analysis for Figure 6A. Treatments showing no overlapped labels (a–c) differ significantly between multiple comparisons (*p* < 0.01–0.0001). Data, mean ± SD (*n* = 3).

MitoMP flow cytometry for oral cancer cells was performed after UVC and/or EANA treatments (Figure 6A). Figure 6B shows that MitoMP-negative (%) of the combined treatment (UVC/EANA) is significantly higher than other single treatments or control in a 24 h incubation.

We next examined the effect of NAC on UVC sensitizing ability of EANA in inducing MitoMP depletion of oral cancer cells. The MitoMP depletion of UVC and/or EANA against oral cancer cells were attenuated by NAC pretreatment (Figure 6B).

### 3.8. Antioxidant Gene Expressions of UVC and/or EANA Treatments in Oral Cancer Cells

**Figure 7:**
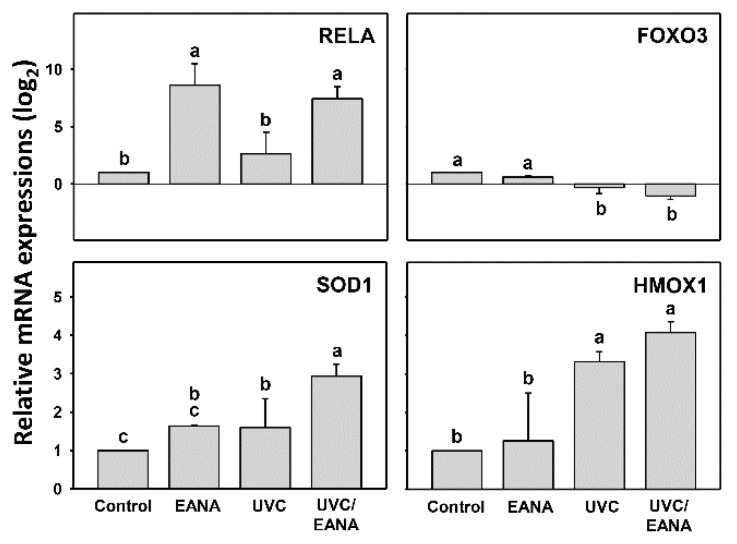
Antioxidant gene expressions of EANA and/or UV treatments for human oral cancer Ca9-22 cells. Cells were treated with control (DMSO only), EANA (5 µg/mL), UVC (12 J/m^2^), and combined treatment (UVC/EANA) for 24 h. Antioxidant mRNA gene expressions such as *RELA*, *FOXO3*, *SOD1*, and *HMOX1* were tested by qRT-PCR analysis. Treatments showing no overlapped labels (a–c) differ significantly between multiple comparisons (*p* < 0.05–0.0001). Data, mean ± SD (*n* = 3).

As shown in above evidence for oxidative stress generation, it is necessary to examine the status for the antioxidant signaling expressions. Figure 7 shows that the *RELA* gene is highly expressed in both EANA and UVC/EANA treatments. Both *SOD1* and *HMOX1* genes show the highest expression in UVC/EANA treatment compared to others, suggesting the UVC/EANA cooperatively induces antioxidant gene expressions for *SOD1* and *HMOX1*. In contrast, the *FOXO3* gene is downregulated in both EANA and UVC/EANA treatments, especially for UVC/EANA treatment.

### 3.9. γH2AX Changes of UVC and/or EANA Treatments in Oral Cancer Cells

**Figure 8:**
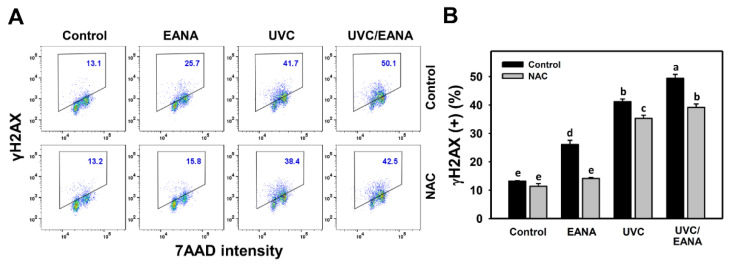
γH2AX analyses of EANA and/or UV treatments for human oral cancer Ca9-22 cells. With or without NAC (10 mM) pretreatment for 1 h, cells were post-treated with control (DMSO only), EANA (5 µg/mL), UVC (12 J/m^2^), and combined treatment (UVC/EANA) for 24 h. (**A**) Typical flow cytometry patterns for γH2AX content. γH2AX intensity-positive regions were marked with a box. (**B**) Statistical analysis for (A). Treatments showing no overlapping labels (a–e) differ significantly between multiple comparisons (*p* < 0.01–0.0001). Data, mean ± SD (*n* = 3).

γH2AX flow cytometry for oral cancer cells after UVC and/or EANA treatments was performed to detect double strand break (DSB) DNA damage (Figure 8A). Figure 8B shows that γH2AX-positive (%) of the combined treatment (UVC/EANA) is significantly higher than other single treatment or control for 24 h incubation.

We next examined the effect of NAC on the UVC sensitizing ability of EANA in inducing γH2AX DNA damage of oral cancer cells. The γH2AX induction of UVC and/or EANA treatments against oral cancer cells were attenuated by NAC pretreatment (Figure 8B)

### 3.10. 8-oxodG Changes of UVC and/or EANA Treatments in Oral Cancer Cells

**Figure 9:**
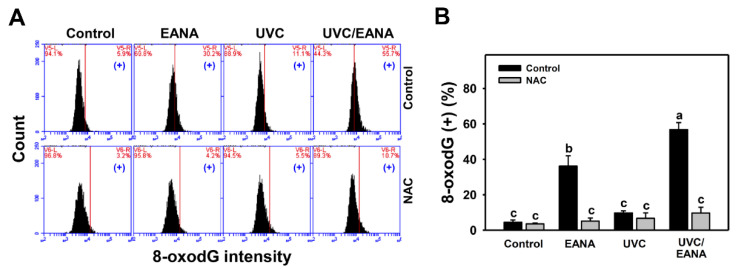
8-oxodG analyses of EANA and/or UV treatments for human oral cancer Ca9-22 cells. With or without NAC (10 mM) pretreatment for 1 h, cells were post-treated with control (DMSO only), EANA (5 µg/mL), UVC (12 J/m^2^), and combined treatment (UVC/EANA) for 24 h. (**A**) Typical flow cytometry patterns for 8-oxodG content. 8-oxodG intensity-positive regions were marked as (+). (**B**) Statistical analysis for (A). Treatments showing no overlapping labels (a–c) differ significantly between multiple comparisons (*p* < 0.0001). Data, mean ± SD (*n* = 3).

8-oxodG flow cytometry for oral cancer cells after UVC and/or EANA treatments was performed to detect oxidative DNA damage (Figure 9A). Figure 9B shows that 8-oxodG-positive (%) of the combined treatment (UVC/EANA) is significantly higher than other single treatments or the control after a 24 h incubation.

We next examined the effect of NAC on the UVC sensitizing ability of EANA in inducing 8-oxodG DNA damage of oral cancer cells. The 8-oxodG induction of UVC and/or EANA against oral cancer cells was attenuated by NAC pretreatment (Figure 9B).

## 4. Discussion

Clinical drugs were developed for anti-oral cancer therapy but some of them showed side effects under higher doses [42]. This drug-induced side effect can become enhanced with the addition of radiation-induced side effects. Therefore, most combined treatments apply a low dose to minimize the possible side effects from drugs and radiation. The present study evaluates the combined treatment effect of low dose EANA (5 μg/mL) and low dose UVC (12 J/m^2^) at 80% oral cancer cell proliferation. Several UVC sensitizing effects and mechanisms for EANA are discussed as follows.

### 4.1. Radiosensitizers with Low Side Effects Are Helpful in Cancer Therapy

Radiosensitizers commonly have side effects. An ideal radiosensitizer is expected to selectively affect cancer cells but to provide low cytotoxic effects on normal cells. This would improve the therapeutic effect of cancer radiotherapy.

UVC was reported to show selective killing of several types of cancer cells but rarely affected normal cells. For example, UVC showed antiproliferation in pancreatic cancer cells but not in normal cells [6]. Similarly, a low dose UVC (14 [10] or 12 J/m^2^ (Figure 1)) showed selective killing to oral cancer Ca9-22 cells with low cytotoxic effects on oral normal HGF-1 cells.

Using selective killing drug compounds, the combined treatment with UVC exhibits potential to become a high performance UVC sensitizer. As reported in our previous work [16], EANA demonstrated a selective killing effect against five different oral cancer cells (IC_50_ values: 8–17 μg/mL) but low cytotoxic to normal oral HGF-1 cells (80% cell proliferation at 16 μg/mL). In the present study, low cytotoxic doses of EANA (5 µg/mL) (providing 80% oral cancer cell proliferation) and UVC show synergistic inhibition of oral cancer cell proliferation, suggesting that EANA is a potential UVC sensitizer and UVC/EANA combined treatment provides a promising anti-oral cancer therapy. Moreover, the air-dried *Nepenthes* plant is a common herb and is available to purchase in herb drug store. Therefore, the cost-effectiveness of EANA is much cheaper than clinical drugs.

### 4.2. Oxidative Stresses Are Synergistically Increased in the Combined Treatment (UVC/EANA) of Oral Cancer Cells

Drugs with ROS modulating effect have potential for antiproliferative treatment against several types of cancer cells [43]. Both high dose UVC [44] and EANA [16] were found to induce intracellular oxidative stress. For example, UVC at 200 J/m^2^ for human epidermoid carcinoma A431 cells [44] induces ROS generation. EANA at doses larger than 8 µg/mL (>IC_50_) induces ROS generation in oral cancer Ca9-22 and CAL 27 cells [16].

Similarly, low dose EANA and low dose UVC at low cytotoxicity were found to generate intracellular oxidative stress. In the current study, Ca9-22 cells irradiated with UVC at 14 [10] or 12 J/m^2^ (Figure 4) maintain around 80% cell proliferation and induce ROS generation compared to the untreated control. Moreover, MitoSOX generation and MitoMP depletion were also induced at low dose EANA and low dose UVC. All oxidative stress changes, such as ROS/MitoSOX generation and MitoMP depletion, are synergistically increased by the combined treatment (UVC/EANA) (Figure 4, Figure 5 and Figure 6).

The redox homeostasis is maintained when it is balanced between cellular oxidative stress and the antioxidant system. Cellular antioxidant signaling proteins may be activated or inactivated in response to oxidative stress. For example, HMOX1 is elevated after exposure to ROS-generating agents [45]. In contrast, FOXO3 activation can eliminate ROS level [46] and therefore FOXO3 inactivation or downregulation can elevate ROS level. In the UV-treated cells, ROS was accumulated and in turn activated RELA (NF-kB)-dependent downstream signaling to activate antioxidant machinery (e.g., SOD2 and HMOX1 expression) [47]. Similarly, SOD1 was activated in UVC-irradiated mice [48]. Consistently, the *RELA* mRNA was highly expressed in both EANA and UVC/EANA treatments. Both *SOD1* and *HMOX1* mRNA showed higher expression in UVC/EANA treatment than others. While, the *FOXO3* mRNA was downregulated in both EANA and UVC/EANA treatments. Accordingly, these antioxidant gene expressions support our finding that oxidative stress was induced after UVC and/or EANA treatments. Therefore, these results suggest that antioxidant signaling pathway is involved in regulating oxidative stress for UVC and/or EANA treatments to oral cancer cells.

### 4.3. DNA Damages Are Synergistically Increased in the Combined Treatment (UVC/EANA) of Oral Cancer Cells

In addition to ROS induction, high dose UVC [49] and EANA [16] can induce DSB DNA damage (γH2AX). Moreover, high dose UVC [50] can induce oxidative DNA damage (8-oxodG). Similarly, low dose UVC and low dose EANA induces DNA damage detected by γH2AX and 8-oxodG flow cytometry. All these DNA damages are synergistically increased in the combined treatment (UVC/EANA).

### 4.4. Apoptosis Is Synergistically Increased in the Combined Treatment (UVC/EANA) of Oral Cancer Cells

Oxidative stress and DNA damage are known to contribute to induce apoptosis. As expected, low dose UVC and low dose EANA induce apoptosis detected by subG1 accumulation, annexin V/7AAD, pancaspase flow cytometry, and Western blotting for apoptosis signaling. All these apoptosis changes are synergistically increased in combined treatment (UVC/EANA) (Figure 2 and Figure 3).

PARP functions in DNA repair and programed cell death and PARP cleavage is a marker for apoptosis [51]. Low dose UVC (10 and 12 J/m^2^, respectively) induces PARP cleavage in human colorectal cancer cells [9] and oral cancer Ca9-22 cells (Figure 3E). In addition to PARP, low dose UVC and low dose EANA increase the expressions for cleaved forms of caspases (c-Cas) 8, 9, and 3. c-Cas 8 shows higher expression than c-Cas 9 suggesting that this extrinsic apoptosis pathway contributes more to apoptosis than to the intrinsic apoptosis pathway. All apoptosis changes of PARP and caspases are synergistically increased in a combined treatment (UVC/EANA) (Figure 3).

### 4.5. Cell Cycle Arresting at G2/M Is Synergistically Increased in the Combined Treatment (UVC/EANA) of Oral Cancer Cells

Low dose UVC and low dose EANA decrease G1 population and increase G2/M population (Figure 2B,C). Extracellular signal-regulated kinase (ERK) plays a vital role in cell cycle regulation, such as G1/S and G2/M [41]. ERK also functions in association with cyclin-dependent inhibitors such as p21 and p27. UVC/EANA shows higher p-ERK expression than other treatments (Figure 2D). However, the G1 population still exists and the G2/M arrest is not complete in UVC/EANA, resulting in weak change for p21 and p27.

### 4.6. All Separate and Synergistic Changes in UVC and/or EANA of Oral Cancer Cells Are Oxidative Stress Dependent

Low dose EANA and/or UVC treatment-induced antiproliferation, apoptosis, oxidative stress, and DNA damage as mentioned above were downregulated by the ROS scavenger NAC. These findings suggest that the UVC sensitizing effect of EANA is dependent on oxidative stress.

## 5. Conclusions

Low dose EANA and low dose UVC exhibit low cytotoxicity to both oral cancer and oral normal cells. Combined treatment (UVC/EANA) preferentially kills the oral cancer cells and does affect normal oral cells less. Mechanistically, antiproliferation, oxidative stress, DNA damage, and apoptosis are synergistically increased in oral cancer cells with a combined treatment in comparison to the other single treatments. All these changes were recovered by NAC pretreatment. With the selective killing properties of low dose EANA and low dose UVC, EANA provides a novel UVC sensitizing agent to improve the antiproliferation of oral cancer treatment without the side effect of cytotoxicity to normal oral cells.

## Figures and Tables

**Table 1:** Primer information for antioxidant-associated genes *.

Genes	Forward Primers (5′→3′)	Reverse Primers (5′→3′)	Length
*RELA*	TCAAGATCTGCCGAGTGAAC [33]	GACGATCGTCTGTATCTGGC	306 bp
*FOXO3*	TACGAGTGGATGGTGCGTT	GGCTTTTCCGCTCTTCC	189 bp
*SOD1*	AGGGCATCATCAATTTCGAGC [36]	CCCAAGTCTCCAACATGCCTC [37]	211 bp
*HMOX1*	CCTTCTTCACCTTCCCCAACAT [37]	GGCAGAATCTTGCACTTTGTTGC [37]	251 bp
*GAPDH*	CCTCAACTACATGGTTTACATGTTCC [38]	CAAATGAGCCCCAGCCTTCT [39]	220 bp

* Primers without reference were designed in this study.
